# Effects of mixed lactic acid bacteria on intestinal microbiota of mice infected with *Staphylococcus aureus*

**DOI:** 10.1186/s12866-018-1245-1

**Published:** 2018-09-06

**Authors:** Dayong Ren, Shengjie Gong, Jingyan Shu, Jianwei Zhu, Hongyan Liu, Ping Chen

**Affiliations:** 1 College of Food Science and Engineering, Jilin Agricultural University, Changchun, 130118 People’s Republic of China; 2 Veterinary Science Department, College of Veterinary Medicine, Jilin University, Changchun, 130062 People’s Republic of China

**Keywords:** Mixed lactic acid bacteria, *Staphylococcus aureus*, Intestinal microbiota, High-throughput sequencing

## Abstract

**Background:**

The stability of intestinal microorganisms plays an important role in human health, as the intestines perform important functions in the human body. *Staphylococcus aureus* is a Gram-positive, facultative anaerobic bacteria, it causes human infection worldwide, and is a major pathogen that causes intestinal infection. Mixed lactic acid bacteria (LAB) may have potential in the prevention and treatment of *S. aureus* infection. In the present study, we examined the effects of mixed LAB treatment on intestinal microbiota modulation in mice infected with *S. aureus*.

**Results:**

High-throughput sequencing of the V3-V4 region of the bacterial 16S rRNA gene showed that the mixed LAB maintained the richness and diversity of the microbiota in the mouse intestine. By establishing operational taxonomic units and using rarefaction analysis, rank-abundance distribution curves, heat maps, Venn diagrams, bacterial community structures, and hierarchical clustering analysis, *Bacteroidales*, *Lachnospiraceae*, *Bacteroides* and *Prevotellaceae* were the most abundant taxa in the samples, we found that the composition of the intestinal microbiota was similar between the protection group administered mixed LAB and the negative control group.

**Conclusions:**

*Staphylococcus aureus* destroys the stable intestinal microbiota structure of mice, treatment with mixed LAB could prevent *S. aureus* infection in mice and improve the structure of the intestinal microbiota.

## Background

The human gut microbiome can be considered our neglected organ [1]. Most human pathogens enter the body through the mucosal surfaces, especially those of the intestine [2]. It is here that over 10^13^ bacteria reside, conferring many benefits to intestinal physiology and forming a truly mutualistic relationship with humans [3, 4]. The structure of intestinal flora is closely related to disease, as when the balance of the intestinal ecosystem is destroyed, it seriously affects human health. For example, interactions among the intestinal microbiota are involved in the pathogenesis of inflammatory bowel disease. Some clinical trials have proved that improving the diversity of the intestinal microbiota is helpful for treating intestinal inflammation [5]. *Staphylococcus aureus* is a Gram-positive, facultative anaerobic bacteria found in a range of hosts, these organisms are found in the intestine [6], which is a kind of bacteria that are harmful to human body. A study found that major species among the obligate microbiota were suppressed in parallel with an increase in the proportion of potentially pathogenic microorganisms, and the latter were *S. aureus* in a third of the cases [7]. In the mid-twentieth century, *S. aureus* was first recognized as a cause of enterocolitis [8], one of the most serious complications of surgery. This infection is accompanied by rapid deterioration. If there is a delay in the initiation of treatment, it can be fatal [9]. Previous research on *S. aureus* infection in mice showed that the initial infection was accompanied by lethargy, lack of appetite, hair towering, and excretory adhesions; continued infection led to subcutaneous pustules, part of the epidermis falling off, reduced tail temperature, and perianal swelling. The internal organs of the diseased mice were black, indicating serious illness [10].

The intestinal microbiota is a positive health asset that crucially influences the normal structural and functional development of the mucosal immune system [11]. The host benefits from symbiotic microbial populations but also needs protection from these symbiotic organisms, since studies have shown that a healthy microbiota requires a balanced relationship between symbionts, commensal organisms, and pathogens [12]. A community-wide balance is important for maintaining healthy gut flora, and alterations in this balance can lead to disease [13]. Commensal bacteria are implicated in digestive tract health and disease. As is well known, the intestinal microbiota plays a role in regulating host cell proliferation and tissue repair [14]. Probiotics play an important role in preventing the overgrowth of potentially pathogenic bacteria and in maintaining the integrity of the gut mucosal barrier [8]. In our previous study, we found that lactic acid bacteria (LAB) protected the internal organs of mice infected with *S. aureus*. The incidence of normal growth weight and increased secretion of secretory IgA in the intestine was significantly higher when the mice were treated with mixed LAB than when they were treated with a single lactic acid bacteria, and the interferon-γ/interleukin-4 ratio decreased significantly from infection to convalescence [10]. To determine whether the improvement of the symptoms of *S. aureus* infection in mice is related to the structure of the intestinal flora and to study the effects of the mixed LAB on the intestinal microbiota of mice infected with *S. aureus*, we used Illumina high-throughput sequencing. Illumina’s MiSeq platform has been shown to be at least as good as the 454 platform and costs much less [15]*.*

## Methods

### Bacterial strains

The *Lactobacillus plantarum* T4 and T8 were both obtained from a farm from different homemade fermented foods. In the present experimental study, the two strains were mixed in the ratio 1:1 to form mixed LAB. Both strains were cultured in de Man-Rogosa-Sharpe broth for 24 h at 37 °C, harvested by centrifugation (4 °C, 7500 rpm, 2 min), and diluted with 0.9% sterile saline. The concentration of probiotics was approximately 10^9^ CFU/mL. *S. aureus* was isolated and preserved at the Laboratory of Toxicology, College of Food Science and Engineering, Jilin Agricultural University. The organism was cultivated with shaking in Luria-Bertani broth for 24 h at 37 °C, and the concentration of *S. aureus* was maintained at 10^6^ CFU/mL. All the bacteria were stored at 4 °C and used for subsequent tests. After the end of the experiment, the bacteria were killed by autoclaving.

### Animals and experimental groups

Female pathogen-free Kunming mice aged 5 weeks and weighing 18–20 g were purchased from the Changchun Institute of Biology Products Co., Ltd. The mice were provided water and food ad libitum and housed under a 12 h light and dark cycle at a suitable temperature and humidity, and they were acclimated for 1 week. The animals were divided into the protection group (P), treatment group (T), and control group. After 1 week of adaptive feeding, the mice in the P group were fed *Lactobacillus* T4, T8, and a mixture of the two strains for 1 week, and then *S. aureus* for 1 week. The mice in the treatment group were then given single and mixed lactic acid bacteria for 1 week. The control group was divided into a negative control group (Con), which was provided adequate water and food and 0.9% sterile saline instead of bacteria, and a positive control group (Mod), in which the mice were infected with *S. aureus* for 1 week without the LAB intervention until they were euthanized. There were six mice in each experimental group. All animal handling and experiments were approved by the Animal Care Ethics committee of Jilin Agricultural University (Changchun, Jilin, China).

### Fecal sample collection, DNA extraction and PCR amplification

Mice were anesthetized by intraperitoneal injection (200 mg/kg/mouse) of 20% arababital and sterile saline solution (1:3 v:v ratio) [10]. The mice were killed by cervical dislocation after anesthesia. On the last day of the experiment, fresh stool samples were collected from the mice, placed in sterile containers, and then frozen at − 80 °C for further analysis [16]. Microbial DNA was extracted from the mouse feces by using the E.Z.N.A.™ stool DNA kit (Omega Bio-tek, Guangzhou Feiyang Biological Engineering Co., Ltd) according to the manufacturer’s protocols [10]. The extracted DNA template was stored at − 20 °C and used in subsequent tests. The samples were sent to Shanghai Meiji Biological Medicine Technology Co. Ltd., and examined by Illumina high-throughput sequencing. The V3-V4 region of the bacterial 16S rRNA gene was amplified by PCR. The forward and reverse primers used were 338F (5′-ACTCCTACGGGAGGCAGCAG-3′) and 806R (5′-GGACTACHVGGGTWTCTAAT-3′), respectively [10, 17].

### Statistical analysis

OTU(Operational taxonomic units) is used in phylogenetic or population genetics research in order to determine the level of similarity among closely related individuals and group them in a certain classification unit (strain, genus, species, group, etc.) [18]. Generally, it uses a similarity level of 97% for statistical analysis of biological information. OTU partitioning of all sequences was performed to enable comparison of the groups, by using the software platform Usearch (version 7.1, http://drive5.com/uparse/). Then, mother was used for rarefaction analysis, and the R language tool was used to generate graphs.

## Results

### Sequencing analysis, richness and diversity of intestinal microbiota in the mice

**Fig. 1:**
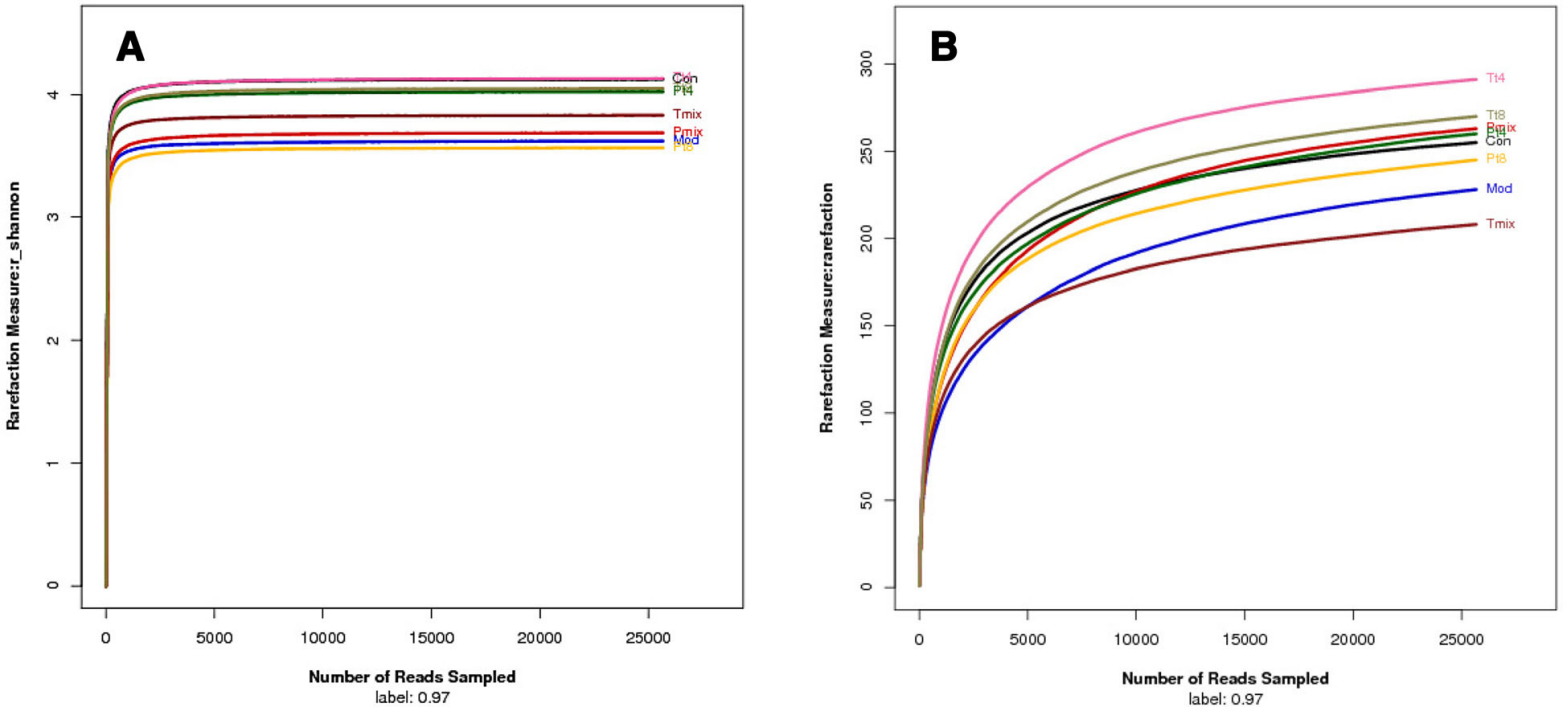
**a** Rarefaction analysis of the microbiota of the mouse intestine. **b** Shannon-Wiener curves for each group. *Con* Control, Mod: Model, *P* Protection, *T* Treatment, *T4/T8* *Lactobacillus plantarum* T4/T8, *Mix* A mix of the T4 and T8 strains

The diversity indices for the intestinal microbiota of the mice were shown in Table 1. The diversity in the Mod group was significantly different from that in the Con group. In contrast, in the P group, the structure of the intestinal flora after treatment with LAB was similar to that in the Con group. The Shannon and rarefaction curves of the intestinal microbiota of the mice in different groups were shown in Fig. 1. The number of OTUs at a 97% distance from the 16S rRNA gene was 255, 228, 260, 245, 263, 291, 270, and 208 OTUs for the Con, Mod, T4 P group, T8 P group, mixed LAB P group, T4 T group, T8 T group, and mixed LAB T group, respectively. The indices of diversity were significantly higher in the Con group than in the Mod group, and the indices of the mixed LAB-treated P group were closer to those in the Con group than the Mod group (Fig. 1a). Rarefaction index analysis (Fig. 1b) indicated that the diversity and richness of the microbial community were the best after treatment with mixed LAB in the P group, followed by the Con group.

**Table 1:** Diversity indices for the microbiota of the mouse intestines

Sample	Reads	OTU	Ace	Chao	Coverage	Shannon	Simpson
Con	25,665	255	270	270	0.999026	4.12	0.0315
Mod	25,665	228	250	248	0.998714	3.62	0.0602
Pt4	25,665	260	282	284	0.998714	4.02	0.0397
Pt8	25,665	245	268	274	0.998753	3.56	0.0721
Pmix	25,665	263	279	276	0.998909	3.69	0.0723
Tt4	25,665	291	305	320	0.998870	4.13	0.0428
Tt8	25,665	270	290	292	0.998831	4.05	0.0356
Tmix	25,665	208	226	233	0.998948	3.83	0.0389

*Con* Control, *Mod* Model, *P* Protection, *T* Treatment, *T4/T8 Lactobacillus plantarum T4/T8 Mix* A mix of the two strains

### Rank-abundance distribution curve and heat map analysis

**Fig. 2:**
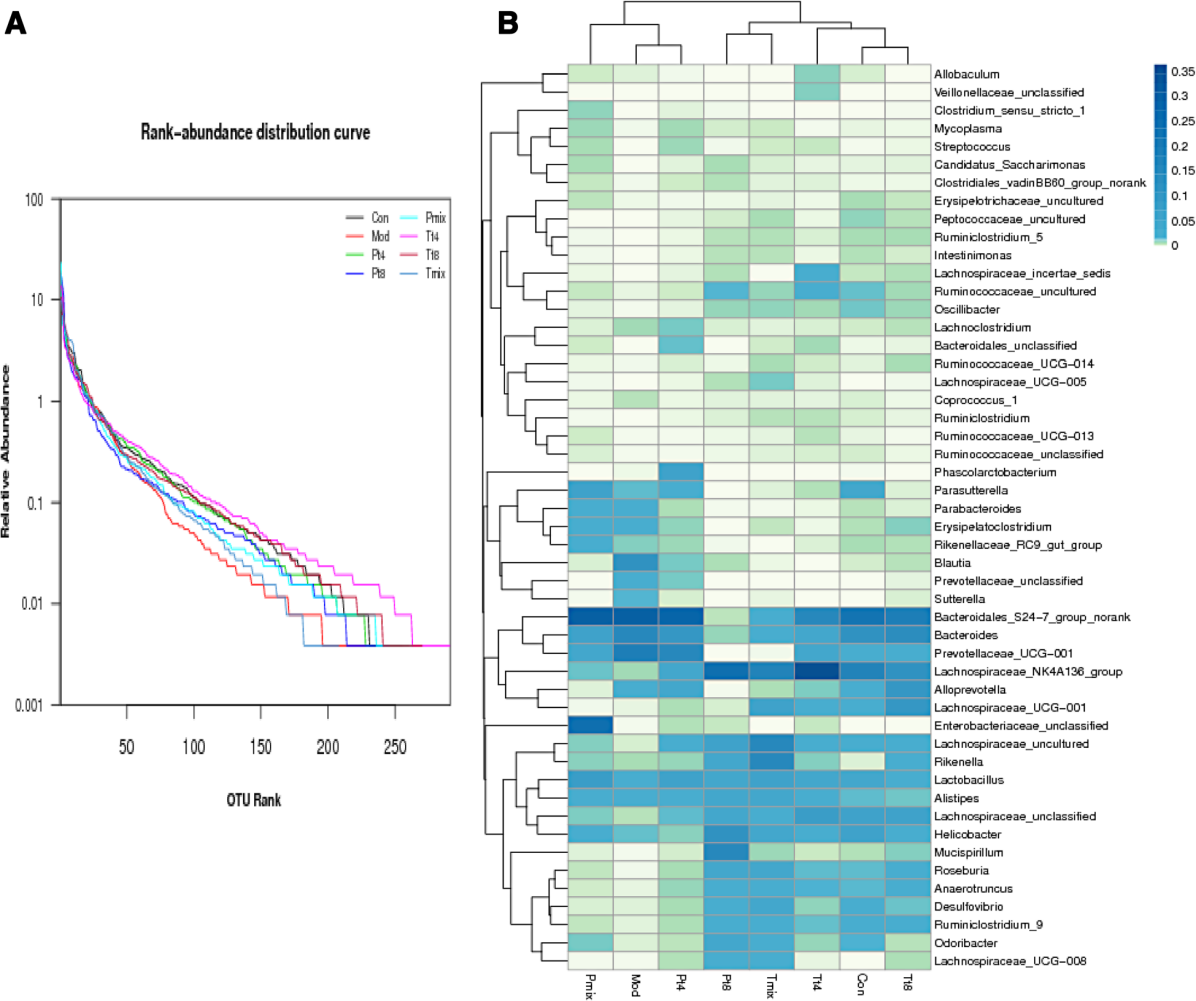
**a** OTU rank-abundance distribution curve. **b** Heat map of specimens showing relative abundance of main identified bacteria at the genus level. *Con* Control, *Mod* Model, *P* Protection, *T* Treatment, *T4/T8* *Lactobacillus plantarum* T4/T8, *Mix* A mix of the T4 and T8 strains

Figure 2 shows the rank-curve distribution curves of the intestinal microbiota in the mice from the various groups in this study. The curves indicate both species abundance and distribution evenness. The higher the abundance of the species, the greater is the range of the curve on the horizontal axis. In contrast, the smoothness of the curve reflects the uniformity of the species in the sample; the gentler the curve, the more uniform is the distribution of species. The heat map directly reflects the data information of different colors [18]. The distribution ranges in the Con group were wider than those in the Mod group, indicating that the species were more abundant in the former than the latter. The wide of distribution range in the P group treated with mixed LAB was closer to that in the control. The curve plot of the Mod group was smaller than that of the Con group; this indicates that the species distribution in the Con group was more even (Fig. 2a). Bacterial abundance at the genus level also significantly differed between the Mod and Con groups; that in the P group treated with mixed LAB was closer to the abundance in the Con group, indicating that these groups have a high degree of similarity (Fig. 2b).

### Distribution of intestinal microbiota in the mice

**Fig. 3:**
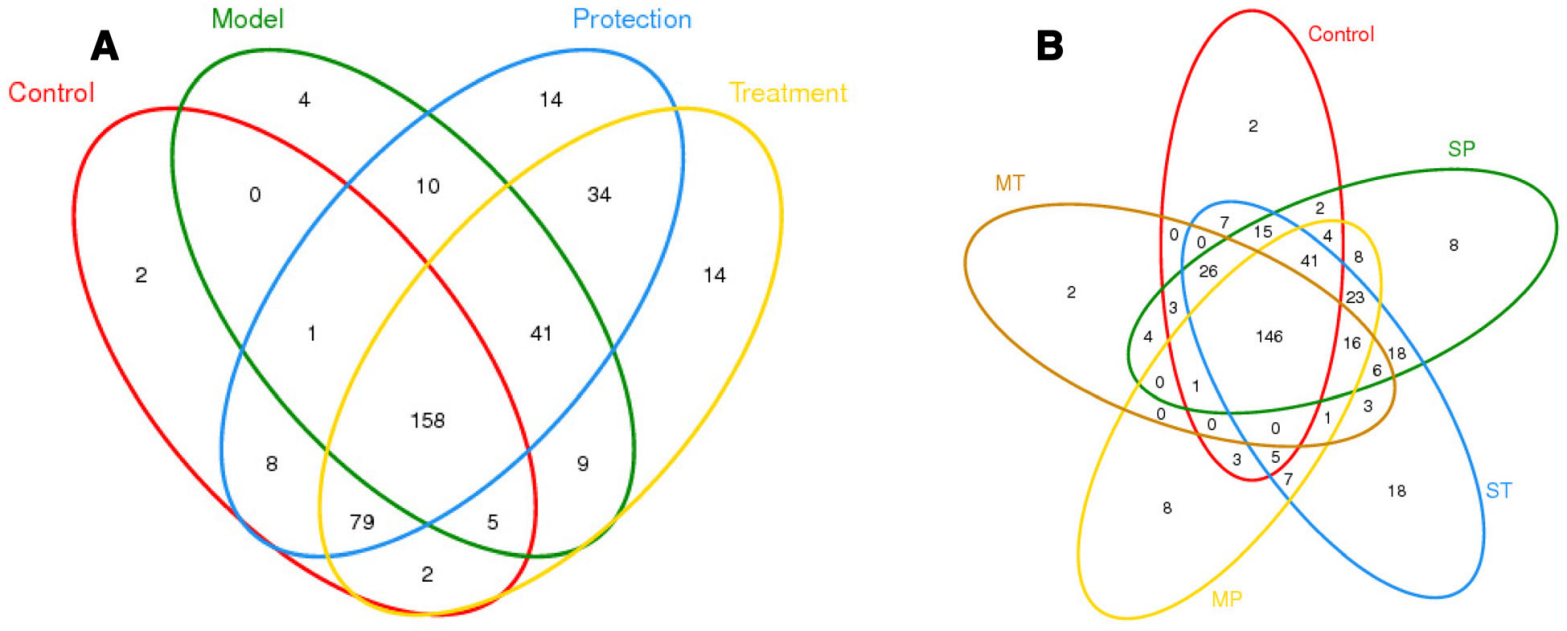
Venn diagram showing the unique and common operational taxonomic units in the mouse intestine. **a** Distribution of intestinal microbiota in the prevention and treatment group. **b** Distribution of intestinal microbiota in the single and mixed LAB group. *S(M)P* Single(mixed)lactic acid bacteria administered to the protection group. *S(M)T* Single(mixed)lactic acid bacteria administered to the treatment group

A Venn diagram was used to show the number of common and unique OTUs, thus intuitively describing sample similarity and overlap [18]. The unique OTUs in the Con, Mod, P, and T groups were 2, 4, 14, and 14, respectively; the total number of OTUs in each of these samples was 255, 228, 345, and 342 respectively. Thus, the ratios of unique to total OTUs were 0.78%, 1.75%, 4.05%, and 4.09%, respectively. The common OTU number in these four groups was 158 (Fig. 3a). The unique OTUs in the Con group, P group administered mixed LAB, T group administered mixed LAB, P group administered a single LAB, and T group administered a single LAB were 2, 8, 2, 8and 18, respectively; the total number of OTUs in each of these samples was 255, 263, 208, 321and 332 respectively. The ratios of the unique OTUs accounting for the total OTUs were 0.78%, 3.04%, 0.96%, 2.49% and 5.42%, respectively. The number of common OTU in these five groups was 146 (Fig. 3b). These findings indicate that the microbiota structure can change because of the intervention of LAB.

### Microbial community structure at the genus level and hierarchical clustering analysis

**Fig. 4:**
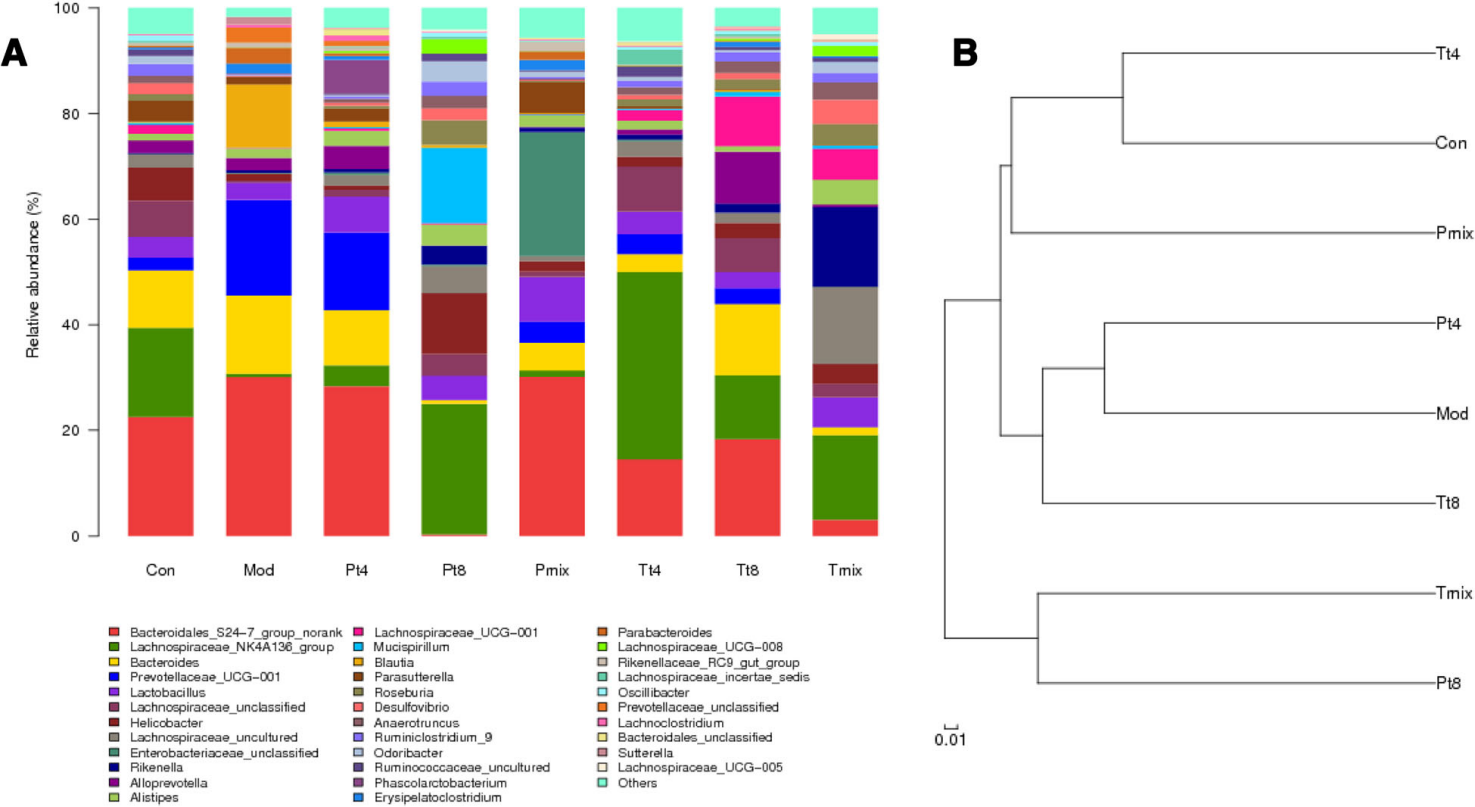
**a** Taxonomic classification of the bacterial communities at the genus levels in the eight mouse groups. **b** Hierarchical clustering analysis of bacterial communities from different groups. The community structure similarity of each sample at the OTU (97%) level. *Con* Control, *Mod* Model, *P* Protection, *T* Treatment, *T4/T8* *Lactobacillus plantarum* T4/T8, *Mix* A mix of the T4 and T8 strains

At the level of the genus, 35 genera were identified (Fig. 4a). *Bacteroidales, Lachnospiraceae,* and *Bacteroides* species were the most prominent in the Con group, while *Prevotellaceae* occurred at a significantly higher proportion in the Mod group than the other groups. The microbial community structure of the P group administered mixed LAB differed from that in the Mod group; it was closer to that in the Con group. The hierarchical clustering analysis (Fig. 4b) confirmed the above results. The similarity of community composition between Mod group and Con group was significantly different. Examination of the effect of mixed LAB on the overall composition of the microbial communities in the mouse intestines showed that the T and P groups administered mixed LAB, the P and Con groups had similar compositions, the T and Con groups had significantly different compositions.

## Discussion

Intestinal microbes have become an indispensable part of human health research. Their contributions to nutrition are multifaceted [19], and they form a symbiotic ecosystem that maintains a homeostatic balance within the human body. However, this equilibrium can be disrupted by pathological conditions interfering with intestinal physiology [20]. Alterations in the morphology, function, and bacterial flora of the intestine have all been reported as causes of inflammation. Pathogenic gut flora might negatively affect patients’ nutrition as well as their metabolic efficiency by reducing the microbiota diversity, which would decrease the production of beneficial metabolites [21]. Studies have shown that the stimulating effect of colon motility is associated with the normalization of intestinal microbiota with probiotics [7], which can improve intestinal and intestinal mucosal reconstruction [22]. For example, strains of *Lactobacillus paracasei* can be used to prevent infection or treat the complications of *S. aureus* infection [8]. In the present study, we investigated the characteristics of intestinal microbiota in mice infected with *S. aureus* and treated with LAB before and after the intervention.

OTU values were obtained using Illumina high-throughput sequencing. The Con and Mod groups were significantly different, and compared to the findings of the T group, those of the P group treated with mixed LAB were better and closer to the findings of the Con group. Research has shown that probiotics, defined as live microorganisms that when administered in a sufficient amount, may have a positive effect on the host microbiota and health benefits for the host, affect the intestinal microbiota [23, 24]. Fermentative LAB are aerobic and are abundant in the environment and in foods, and they comprise a major part of the commensal flora in the small intestine [25]. Therefore, treatment with LAB can effectively prevent the destruction of the structure of intestinal flora and protect the intestinal tract against pathogens. Further, mixed LAB are more effective at preventing infection than treating it.

The diversity and abundance of bacterial species are thought to be aspects of a healthy gut microbiome [26]. In the present study, the four most abundant taxa were *Bacteroidales*, *Lachnospiraceae*, *Bacteroides* and *Prevotellaceae*. *Bacteroidales* are strict anaerobes, and members of the *Bacteroidales* group have recently been suggested as indicators of fecal contamination [27]. Additionally, members of the *Lachnospiraceae* family are known to participate in the fermentation of carbohydrates in the human gastrointestinal tract [28]*. Bacterioides thetaiotamicron* is a Gram-negative anaerobe and a prominent part of the distal intestine microbiota in mice and humans; it comprises 12% of all *Bacteroides* species in the human intestine and modulates several essential host functions [29]. *Prevotella* species like *Prevotella pallens* produces acids (acetic and succinic acids) from glucose, maltose, and sucrose [30]. Organisms of the taxon *Prevotellaceae* accounted for 18.11% of the microbiota in the Mod group. In the P group, *Prevotellaceae* from the *Lactobacillus* T4 culture accounted for 14.61% of the total sequences, a proportion close to that in the Mod group. In other analyses as well, T4-treated mice had a similar intestinal flora structure to mice in the Con group, but the proportion of *Prevotella* in the community analysis was closer to that in the Mod group, indicating that single colonies of T4 were not able to regulate the structure of intestinal flora to reach the normal state.

Our results showed that the use of mixed LAB to prevent or control *S. aureus* infection yielded the best results in terms of improvements to the structure of intestinal flora. Studies have shown that the proportion of the intestinal microbiota differs between healthy individuals and those who are not. The diversity of the intestinal microbiota in patients with chronic kidney disease is lower, and the LAB strains affect liver and kidney function by changing the composition of the microbiota [31]. We previously found that the structure of the intestinal flora of mice treated with mixed LAB was restored and the symptoms of *S. aureus* infection improved [10], indicating that intestinal health was associated with disease recovery.

## Conclusion

In summary, compared to the T group treated with mixed LAB, the P group treated with LAB had a more similar bacterial community structure to that of the Con group. This finding suggested that mixed LAB are better at protecting against *S. aureus* infection than treating it. Further, intestinal structure can recover to normal with this treatment, and the symptoms of *S. aureus* infection in mice improve.

## Abbreviations

Con: negative control group
LAB: Lactic acid bacteria
Mod: positive control group
OTU: Operational taxonomic unit
P: Protection group
T: Treatment group

## References

[1] Ehrlich SD (2016). The human gut microbiome impacts health and disease. Comptes Rendus Biologies.

[2] Magalhaes JG, Tattoli I, Girardin SE (2007). The intestinal epithelial barrier: how to distinguish between the microbial flora and pathogens. Semin Immunol.

[3] O'Shea EF, Cotter PD, Stanton C, Ross RP, Hill C (2012). Production of bioactive substances by intestinal bacteria as a basis for explaining probiotic mechanisms: bacteriocins and conjugated linoleic acid. Int J Food Microbiol.

[4] Rakoffnahoum S, Paglino J, Eslamivarzaneh F, Edberg S, Medzhitov R (2004). Recognition of commensal microflora by toll-like receptors is required for intestinal homeostasis. Cell.

[5] Feng XB, Jiang J, Min L, Gang W, You JW, Jian Z (2016). Role of intestinal flora imbalance in pathogenesis of pouchitis. Asian Pac J Trop Med.

[6] Chiefari AK, Perry MJ, Kellycirino C, Egan CT (2015). Detection of *Staphylococcus aureus* enterotoxin production genes from patient samples using an automated extraction platform and multiplex real-time PCR. Mol Cell Probes.

[7] Khalif IL, Quigley EM, Konovitch EA, Maximova ID (2005). Alterations in the colonic flora and intestinal permeability and evidence of immune activation in chronic constipation. Dig Liver Dis.

[8] Bendali F, Madi N, Sadoun D (2011). Beneficial effects of a strain of *Lactobacillus paracasei* subsp. *paracasei* in *Staphylococcus aureus*-induced intestinal and colonic injury. International journal of infectious diseases Ijid official publication of the international society for. Infectious Diseases.

[9] Takahata M, Sugiura Y, Ameyama S, Yotsuji A, Mitsuyama J, Sumiyama Y, Kusachi S (2004). Influence of various antimicrobial agents on the intestinal flora in an intestinal MRSA-carrying rat model. J Infect Chemother.

[10] Ren D, Gong S, Shu J, Zhu J, Rong F, Zhang Z, Wang D, Gao L, Qu T, Liu H, Chen P (2017). Mixed *Lactobacillus plantarum* strains inhibit *Staphylococcus aureus* induced inflammation and ameliorate intestinal microflora in mice. Biomed Res Int.

[11] O'Hara AM, Shanahan F (2006). The gut flora as a forgotten organ. EMBO Rep.

[12] Mcloughlin RM, Mills KHG (2011). Influence of gastrointestinal commensal bacteria on the immune responses that mediate allergy and asthma. J Allergy Clin Immunol.

[13] Kang DW, Jin GP, Ilhan ZE, Wallstrom G, Labaer J, Adams JB, Krajmalnikbrown R (2013). Reduced incidence of *Prevotella* and other fermenters in intestinal microflora of autistic children. PLoS One.

[14] Liu HX, Rocha CS, Dandekar S, Wan YY (2016). Functional analysis of the relationship between intestinal microbiota and the expression of hepatic genes and pathways during the course of liver regeneration. J Hepatol.

[15] Feng Z, Wu C, Zhou J, Wu F, Li J, Li T, Yin Y (2015). Disturbance of the intestinal microbial community by ursolic acid contributes to its function as a regulator of fat deposition. J Funct Foods.

[16] Xiao LL, Hua NR, Shi YL (2017). Early detection of Trichinella spiralis, DNA in the feces of experimentally infected mice by using PCR[J]. Acta Trop.

[17] Lu L, Wan Z, Luo T (2018). Polystyrene microplastics induce gut microbiota dysbiosis and hepatic lipid metabolism disorder in mice[J]. Sci Total Environ.

[18] Cheng Z, Hu X, Sun Z (2016). Microbial community distribution and dominant bacterial species analysis in the bio-electrochemical system treating low concentration cefuroxime. Chem Eng J.

[19] Preidis GA, Ajami NJ, Wong MC, Bessard BC, Conner ME, Petrosino JF (2015). Composition and function of the undernourished neonatal mouse intestinal microbiome. J Nutr Biochem.

[20] Lin P, Ding B, Feng C, Yin S, Zhang T, Qi X, Lv H, Guo X, Dong K, Zhu Y (2017). *Prevotella* and *Klebsiella* proportions in fecal microbial communities are potential characteristic parameters for patients with major depressive disorder. J Affect Disord.

[21] Pasini E, Aquilani R, Testa C, Baiardi P, Angioletti S, Boschi F, Verri M, Dioguardi F (2015). Pathogenic gut Flora in patients with chronic heart failure. Jacc Heart Failure.

[22] Mima K, Ogino S, Nakagawa S (2017). The role of intestinal bacteria in the development and progression of gastrointestinal tract neoplasms.[J]. Surg Oncol.

[23] Reiff C, Kelly D (2010). Inflammatory bowel disease, gut bacteria and probiotic therapy. Int J Med Microbiol.

[24] Xie Q, Pan M, Huang R, Tian X, Tao X, Shah NP, Wei H, Wan C (2016). Short communication: modulation of the small intestinal microbial community composition over short-term or long-term administration with *Lactobacillus plantarum* ZDY2013. J Dairy Sci.

[25] Kawashima T, Kosaka A, Yan H, Guo Z, Uchiyama R, Fukui R, Kaneko D, Kumagai Y, You DJ, Carreras J (2013). Double-stranded RNA of intestinal commensal but not pathogenic Bacteria triggers production of protective interferon-β. Immunity.

[26] Wang W, Cao J, Li JR, Yang F, Li Z, Li LX (2015). Comparative analysis of the gastrointestinal microbial communities of bar-headed goose (Anser indicus) in different breeding patterns by high-throughput sequencing. Microbiol Res.

[27] Liang Z, He Z, Zhou X, Powell CA, Yang Y, Roberts MG, Stoffella PJ (2011). High diversity and differential persistence of fecal *Bacteroidales* population spiked into freshwater microcosm. Water Res.

[28] Paturi G, Butts CA, Bentley-Hewitt KL, Hedderley D, Stoklosinski H, Ansell J (2015). Differential effects of probiotics, prebiotics, and synbiotics on gut microbiota and gene expression in rats. J Funct Foods.

[29] Zocco MA, Ainora ME, Gasbarrini G, Gasbarrini A (2007). *Bacteroides thetaiotaomicron* in the gut: molecular aspects of their interaction. Dig Liver Dis.

[30] Xu S, Lu W, Liu Y, Ming Z, Liu Y, Meng R, Wang H (2016). Structure and diversity of bacterial communities in two large sanitary landfills in China as revealed by high-throughput sequencing (MiSeq). Waste Manag.

[31] Dong Y, Yu X, Wu Y, Chen X, Wei H, Shah NP (2016). Enhancing flora balance in the gastrointestinal tract of mice by lactic acid bacteria from Chinese sourdough and enzyme activities indicative of metabolism of protein, fat, and carbohydrate by the flora. J Dairy Sci.

